# Breathing sounds analysis system for early detection of airway problems in patients with a tracheostomy tube

**DOI:** 10.1038/s41598-023-47904-0

**Published:** 2023-11-29

**Authors:** Hyunbum Kim, Daeyeon Koh, Yohan Jung, Hyunjun Han, Jongbaeg Kim, Younghoon Joo

**Affiliations:** 1 Department of Otorhinolaryngology-Head and Neck Surgery, College of Medicine, The Catholic University of Korea, 2 Sosa-dong, Wonmi-gu, Bucheon, Kyounggi-do 14647 Republic of Korea; 2 School of Mechanical Engineering, Yonsei University, 50 Yonsei-Ro, Seodaemun-Gu, Seoul, 03722 Republic of Korea

**Keywords:** Biotechnology, Health care, Medical research

## Abstract

To prevent immediate mortality in patients with a tracheostomy tube, it is essential to ensure timely suctioning or replacement of the tube. Breathing sounds at the entrance of tracheostomy tubes were recorded with a microphone and analyzed using a spectrogram to detect airway problems. The sounds were classified into three categories based on the waveform of the spectrogram according to the obstacle status: normal breathing sounds (NS), vibrant breathing sounds (VS) caused by movable obstacles, and sharp breathing sounds (SS) caused by fixed obstacles. A total of 3950 breathing sounds from 23 patients were analyzed. Despite neither the patients nor the medical staff recognizing any airway problems, the number and percentage of NS, VS, and SS were 1449 (36.7%), 1313 (33.2%), and 1188 (30.1%), respectively. Artificial intelligence (AI) was utilized to automatically classify breathing sounds. MobileNet and Inception_v3 exhibited the highest sensitivity and specificity scores of 0.9441 and 0.9414, respectively. When classifying into three categories, ResNet_50 showed the highest accuracy of 0.9027, and AlexNet showed the highest accuracy of 0.9660 in abnormal sounds. Classifying breathing sounds into three categories is very useful in deciding whether to suction or change the tracheostomy tubes, and AI can accomplish this with high accuracy.

## Introduction

More than 110,000 tracheostomies are performed each year in the United States^1^. Additionally, as the older population increases, more tracheostomies are performed. Parker et al. reported that respiratory failure, ineffective cough, neurological injury, and carcinoma were increasing in older people and driving the increase in the number of tracheostomies^2^. Although performing a tracheostomy itself must be done very carefully, meticulous management is also necessary after the tracheostomy. Postoperative complications include such as hemorrhage, subcutaneous emphysema, tube decannulation, and tube obstruction^3^, which are life-threatening. Among these complications, tube obstruction can occur at any time after tracheostomy. Das et al. reported that tracheostomy-related death can occur at any time due to tube obstruction in pediatric patients^4^. The causes of tube obstruction include excessive sputum production and insufficient air filtering, but the most important cause is insufficient humidification. To prevent tube obstruction, humidification is essential, and proper suction is also necessary. Despite the humidity, if sputum is not removed and phlegm accumulates, it can block the tube. However, suctioning too frequently is also undesirable because the suction stimulates the trachea wall, which causes contractions and can lead to hypoxia^2^. For these reasons, sputum removal needs to be performed gently at an adequate time. However, obtaining real-time status information about tracheostomy tubes and the airways of tracheostomy patients can be challenging due to the insufficient availability of medical devices, such as fiberscopes and other necessary equipment, which also makes it very difficult to determine the appropriate time for suction. Experienced medical staff can determine when to suction simply by listening to the breathing sounds of the patient, but inexperienced medical staff, particularly caregivers, often miss the appropriate timing for suction. This means that with a medical device capable of analyzing breathing sounds at the same level as experienced medical staff, it is possible to identify the degree of airway obstruction.

Artificial intelligence (AI) is being widely incorporated into healthcare. Many symptoms and diseases are now analyzed and even diagnosed by AI, mainly using images^5,6^, but some trials have also used sounds^7–10^. Shi et al.^7^ reported that they used AI to classify sputum sounds from patients with intubation tubes. Srivastava et al.^8^ reported that AI helped detect chronic obstructive pulmonary disease using respiratory sounds. Nakano et al.^10^ reported that AI helped to detect sleep apnea through tracheal sounds.

Comprehensively, we hypothesized that we could use AI to identify airway problems, including airway obstruction, by analyzing breathing sounds from tracheostomy patients with a microphone. This study aimed to achieve a more granular classification of breathing sounds and attempt to classify them using AI.

## Methods

### Patients

This prospective study was performed at a single university center from March 2021 to April 2022 among patients who received an elective tracheostomy. All tracheostomies were performed by a single surgeon. Under general anesthesia, a horizontal incision was made on the skin at the level of the isthmus. The isthmus was cut after dividing the strap muscles, and a window was created at the second trachea ring. Patients who needed ventilator unit care after tracheostomy or underwent an emergency tracheostomy in the emergency room or intensive care unit (ICU), patients younger than 20 years, and pregnant patients were excluded. Using those criteria, 23 patients with tracheostomy were enrolled in this study. We obtained the following clinical information for all patients. The study was approved by Bucheon St. Mary Hospital of the Catholic University of Korea institutional review board (IRB) (The physiologic changes of trachea according to the degree of sputum after tracheostomy, HC20ONSI0106, approved November 10, 2020). Procedures were followed by IRB ethical standards and the Helsinki Declaration of 1975.

### Recording system

**Figure 1:**
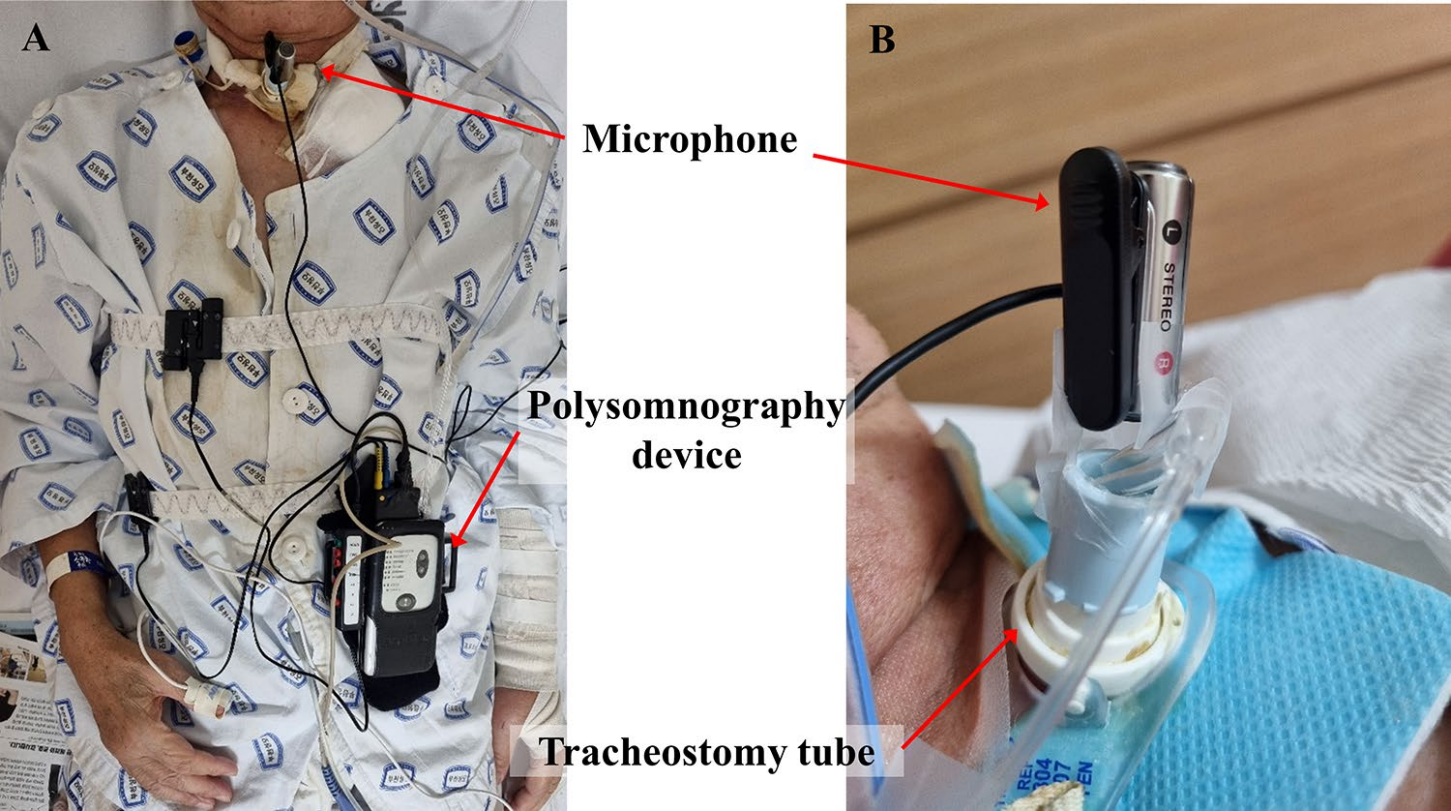
A photograph of the devices installed on the patient with a tracheostomy. A condenser microphone with a voice recorder and parts of a polysomnogram were set on the patient. A nasal prong connected with a polysomnogram was located at the entrance of the tracheostomy tube. A condenser microphone was located at a distance of 2–3 cm from the outer opening of the tracheostomy tube. Pulse oximetry, thoracic and abdominal bands were also set on the patient.

Breathing sound samples were recorded with a voice recorder (Model PCM-A10; Sony, Japan) using a condenser microphone (Model ECM-CS3; Sony, Japan) located two or three cm from the outer opening of the tracheostomy tube in line with the direction of the tube. The recording type was linear pulse code modulation, which can record original sounds without compression. All participants also wore parts of a polysomnography device during the study. The oronasal airflow device was located at the opening of the tracheostomy tube and did not interfere with breathing or recording. However, electroencephalogram, electrooculogram, and electromyography sensors were not set up. A photograph of the devices installed on each participant is shown in Fig. 1.

### Data collection and classification

All data collection started in the ICU immediately after surgery. In general, women are typically inserted with a size 6 or 7 tube, while men are typically inserted with a size 7 or 8 tube. After consulting with the anesthesiologists and considering the height, weight, and pulmonary function of participants, it was determined to use a size 7 tube (TRACOE twist 306-7; GmbH, German). Participants were transferred to the general ward two or three days after tracheostomy, and the existing tracheostomy tube was changed with a new fenestration-type tracheostomy tube (TRACOE twist 304-7; GmbH, German) three to five days after surgery. We collected data only until the tube was changed. As a result, all participants were recorded for an average of 12–16 h a day for 1–5 days after tracheostomy.

Breathing sounds with severe background noise and very low breathing sounds that were not detected in the spectrogram due to some reasons such as sleep were excluded. Breathing sounds during the period when the participants expressed severe dyspnea were also excluded. Even if participants were aware of sputum in their airways, breathing sounds were included if they did not require medical intervention.

Breathing sounds were classified through three stages. First, two expert otorhinolaryngology doctors with more than 10 years of experience listened to all the recording samples and selected the breathing sounds that satisfied the inclusion criteria. Second, all selected breathing sounds were converted into spectrograms and analyzed using the waveforms of the spectrogram. An audio spectrogram is a two-dimensional image that simultaneously presents sound waveforms and spectra. By representing continuously changing spectra as a data sample, spectrograms provide rich audio information and are widely used in deep learning frameworks based on image classification^10–12^. The breathing sound samples were converted into spectrograms using a short-time Fourier transform. A more detailed process of conversion is presented in Supplementary 1.

**Figure 2:**
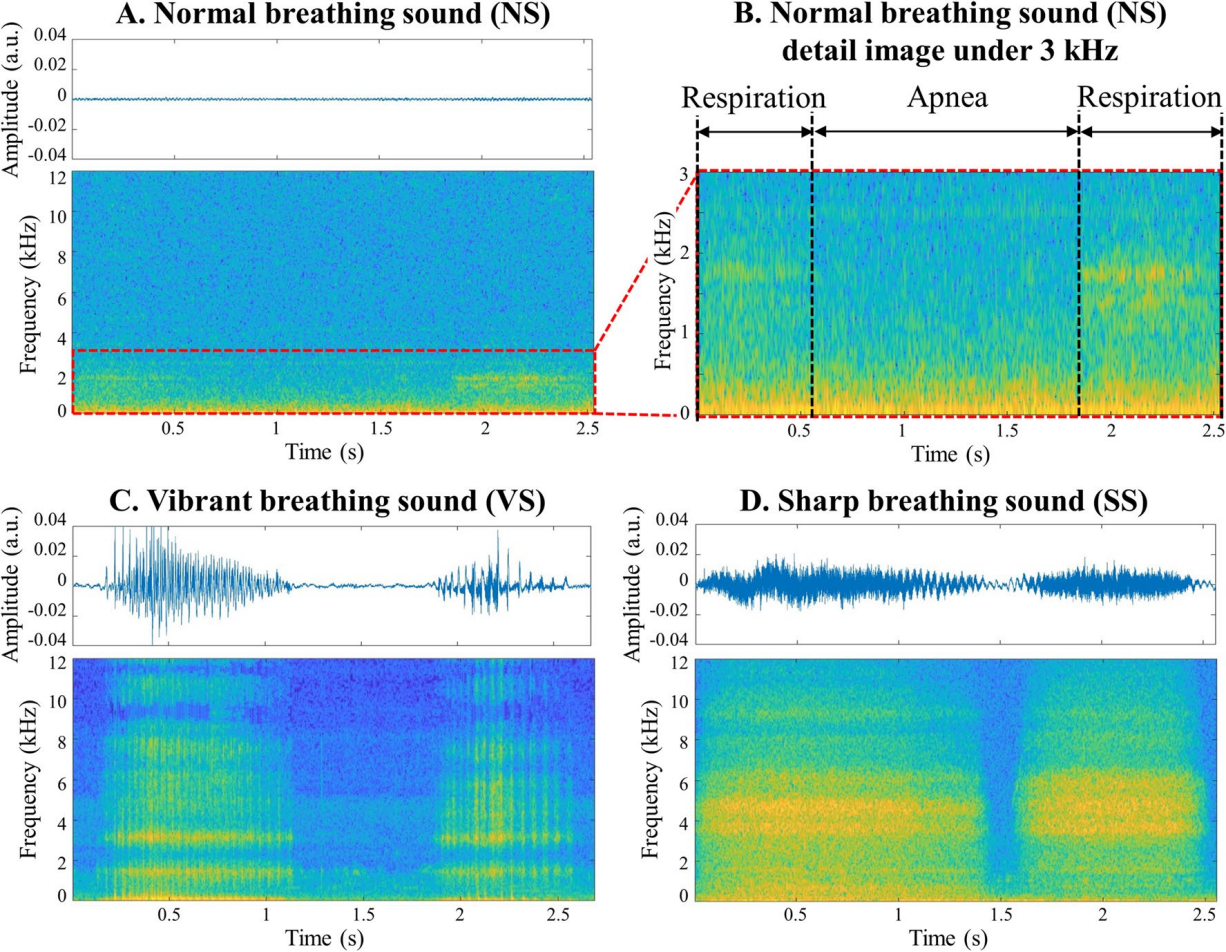
Time domain sound characteristics and spectrograms of breathing sounds. (**A**) The acoustic energy was relatively small and concentrated mainly below 2000 Hz in normal breathing sounds. (**B**) A detailed image of normal breathing sounds under 3 kHz. (**C**) The acoustic energy was scattered over a large area, from 500 to 12,000 Hz in abnormal breathing sounds. In addition to the preceding reason, characteristic multiple vertical lines created by the movable obstacle repeatedly blocking the transmission of sounds were shown in vibrant breathing sounds. (**D**) In sharp breathing sounds, characteristic multiple horizontal lines were shown because fixed obstacles, which induced a wide range of high-frequency breathing sounds, narrowed the airway continuously.

All the breathing sounds were primarily classified into three categories based on the spectrogram waveform: normal breathing sound (NS); low-frequency vibrant breathing sound (VS) that indicates a movable obstacle such as sputum in the tracheostomy tube that requires suctioning; high-frequency sharp breathing sound (SS) that indicates a fixed obstacle, including crusts, and blood clots in the tracheostomy tube that requires suctioning or changing the inner cannula of the tube. Examples of the time-domain wave characteristics and spectrograms of each breathing sound are shown in Fig. 2. In NS, because the airway had no obstacles and minimal friction, the acoustic energy was relatively low and mainly below 2000 Hz. This became evident when examining the spectrogram zoomed below 3000 Hz. There are two primary types of noise. Background noise, which occurs without any specific event, is predominantly found below 1000 Hz. In contrast, noise resulting from events such as speech is most prominent below 1500 Hz. NS, on the other hand, exhibits its energy concentration in the 1500–2000 Hz range. The acoustic energy of the abnormal breathing sounds exhibited a broader acoustic energy distribution, spanning from 500 to 12,000 Hz because the obstacles generated sounds of various frequencies. VS exhibited a repetitive pattern occurring approximately around 100 times per second during respiration indicating the presence of a movable obstacle blocking the trachea or tracheostomy tube. This pattern appears as multiple vertical lines in the spectrogram. In contrast, SS occurred when stiff or fixed obstacles narrowed the cross-section of the airway, which induced a wide range of high-frequency breathing sounds that were more continuous than VS. The pattern of SS appears as multiple horizontal lines in the spectrogram (Fig. 2). There were instances where samples exhibited both VS and SS patterns of the spectrogram. In such cases, they were classified as VS for clinical reasons. First, fixed obstacles are typically composed of sputum or blood clots, which are movable obstacles^3^. Therefore, being in an intermediate stage before becoming entirely SS, they tend to display features closer to VS. Second, suction is a more rapid and easily accessible approach than tube change. Tube change can be performed by only medical staff and carries a higher risk of tracheostomy tube displacement if done within a week after tracheostomy^13^. In contrast, suction can be carried out by caregivers, and there is also a chance for medical staff to reassess the airway after removing all movable obstacles through suction. Additionally, the extent to which a suction catheter can enter the tracheostomy tube itself serves as one of the methods to assess tube obstruction. If fixed obstacles are removed along with movable obstacles during suction, airway problems can often be entirely resolved with suction alone. In cases of a dual cannula of the tracheostomy tube, regardless of the presence of fixed obstacles in the inner cannula, suction should be performed after. For these reasons, the cases displaying both VS and SS patterns of the spectrogram were classified as VS.

Third, two experts classified the breathing sounds according to the spectrogram results. Only when both experts agreed on the result of the spectrogram was it included.

### Methods for AI-based analysis

In this study, we converted breathing sound samples into a spectrogram and Mel frequency cepstral coefficient (MFCC), which are audio features widely utilized to analyze respiratory status. The details of the converted features are described in the following sections. All data processing for sound classification by multiple AI algorithms, such as spectrogram conversion and MFCC extraction, was performed in MATLAB 2019a.

#### MFCC extraction

The MFCC is a group of audio parameters suitable for human auditory characteristics and has been widely applied for speech recognition^14,15^ and respiratory diagnosis^16–18^. In this study, we used MFCC as a tracheal breathing sound feature for machine learning–based classifiers. The MFCCs were extracted in the same frequency range as spectrograms, and the coefficients were obtained for each frame in two variants: MFCC (20) and MFCC (40). A more detailed process of MFCC extraction, the designed filter banks, and examples of extracted MFCCs are presented in Supplementary 2, 3 and 4.

#### MFCC-based machine learning classification methods

For MFCC-based breathing sound classification, a support vector machine (SVM)^19,20^ and k-nearest neighbor (kNN)^21,22^, which are widely used for health status diagnosis using MFCC, are employed. All breathing sound classification for the machine learning algorithms was performed using a desktop machine with an Intel i5-10500F CPU and NVIDIA GeForce RTX1660 Ti (6 GB) GPU. A more detailed process of extraction is presented in Supplementary 5.

#### Spectrogram-based deep learning method: convolutional neural network (CNN)

CNN is a widely and successfully established deep learning algorithm in the field of image classification and pattern recognition^23^. The basic structure of a CNN is the convolution layer, which calculates the tensor transferred to the next layer through a convolution computation between the tensor and the kernel. A CNN topology includes many convolution layers designed based on factors such as kernel size and number. A CNN provides a framework for learning the features common among images in a data group without requiring manual data extraction, and it can generate accurate pattern recognition or image classification–trained models. Biomedical signal classification studies have been accelerated by imaging and learning with CNNs^10–12^. For example, CNN classification based on spectrograms has increased the accuracy of respiratory pattern classification in many clinical fields. In this study, we applied spectrograms converted from one cycle of respiratory data to the following CNN topologies: AlexNet^24^, VGGNet^25^, ResNet^26^, Inception_v3^27^, and MobileNet^28^. The CNN classification was conducted with Lenovo Intelligent-Computing-Orchestration with a batch size of 32 and a maximum iteration of 200,000.

### Ethical approval and consent to participate

The study was approved by the institutional review board (IRB) of Bucheon St. Mary Hospital of the Catholic University of Korea. The approval number is HC20ONSI0106. All participants were provided with an explanation of the study and gave their informed consent. Consent documents were obtained from all participants.

## Results

### Patients

We enrolled and analyzed 23 patients in this study, of whom 21 were male. The average age of all patients was 66 years. Six patients were never smokers, and seven patients were heavy smokers with a history of 30 pack years or more. No patient had a history of asthma, but one patient had been treated for tuberculosis prior. Forced vital capacity and forced expiratory volume at 1 s were measured in 15 patients. Four patients were diagnosed with chronic obstructive pulmonary disease, one patient was diagnosed with restrictive pulmonary disease, and one patient was diagnosed with mixed-type pulmonary disease. Detailed patient information is provided in Table 1.

**Table 1:** Clinical characteristics of the 23 patients with a tracheostomy.

Characteristics	Number of patients (%) or mean (minimum, maximum)
Sex	
Male	21 (91.3)
Female	2 (8.7)
Age (years)	66 (53,79)
Height (cm)	163 (152,175)
Weight (kg)	65 (52,81)
Cause of disease	
Cancer	21 (91.3)
Goiter	1 (4.3)
Subglottic stenosis	1 (4.3)
Smoking	
Never	6 (26.1)
≤ 30 pyrs	10 (43.5)
> 30 pyrs	7 (30.4)
Hypertension	10 (43.5)
Diabetes	2 (8.7)
Cardiovascular disease	2 (8.7)
Asthma	0 (0)
Tuberculosis treatment history	2 (8.7)
Previous operation history in head and neck region	2 (8.7)
Radiotherapy history	1 (4.3)
FEV1/FVC	
≥ 70%	10 (43.5)
≥ 60% and < 70%	4 (17.4)
< 60%	1 (4.3)
No data	8 (34.8)
FVC/Predicted FVC	
≥ 80%	13 (56.5)
< 80%	2 (8.7)
No data	8 (34.8)
Pulmonary disease	
Normal	9 (39.2)
Restrictive	1 (4.3)
Chronic obstructive	4 (17.4)
Mixed	1 (4.3)
No data	8 (34.8)

*pyrs* pack years; *FVC* forced vital capacity; *FEV1* forced expiratory volume at 1 s.

### Analysis of the breathing sounds

A total of 3950 breathing sounds from 378 events in 23 participants were analyzed by segmenting the respiratory cycles in the recording samples. Considering the changes in the breathing cycle over time, we concluded that analyzing only one breathing sound per event would be insufficient. This approach led us to select breath samples primarily based on time. Consequently, we opted to analyze approximately one minute of breathing sounds for each event. After tracheostomy, patients typically breathe at an average rate of 10–11 breaths per minute, resulting in the acquisition of 3950 samples across an average of 378 events. Although both the participants and the medical staff next to them judged that there was no problem in the airway of the participants during the collection of breathing sounds, the breathing sounds were classified into three categories after analysis using a microphone-based recording system and the waveform of the spectrogram. The number and percentage of NS, VS, and SS samples were 1449 (36.7%), 1313 (33.2%), and 1188 (30.1%), respectively. The mean time of each respiration cycle was 3.801 ± 1.592, 3.033 ± 0.949, and 3.876 ± 1.246 s, respectively. Detailed information for each breathing sound is given in Table 2.

**Table 2:** Characteristics of the 3950 breathing sounds from 23 patients.

	Normal sound (NS)	Vibrant sound (VS)	Sharp sound (SS)	Total
Number of breathing sounds (%)	1449 (36.7)	1313 (33.2)	1188 (30.1)	3950 (100)
Number per person (mean ± SD)	63 ± 38.8	57.1 ± 57.0	51.6 ± 43.9	171.7 ± 75.0
Time per 1 cycle of respiration (mean ± SD) (sec)	3.801 ± 1.592	3.033 ± 0.949	3.876 ± 1.246	3.484 ± 1.292

All breathing sounds were segmented by respiratory cycle and classified into three categories: normal breathing sounds (NS), vibrant breathing sounds (VS), and sharp breathing sounds (SS).

### Accuracy of automatic detection of breathing sound samples

**Figure 3:**
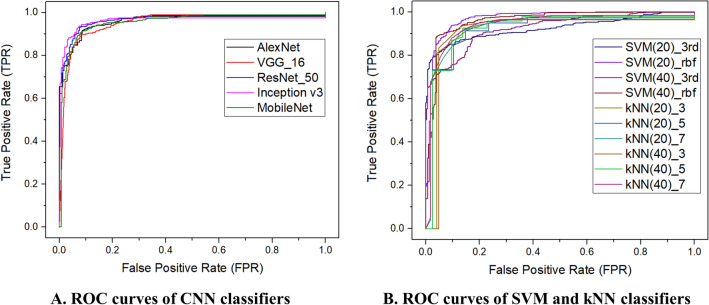
ROC curves of each classifier in the binary classification of normal and abnormal breathing. (**A**) The results of classifiers based on convolution neural networks (CNN) were shown. (**B**) The results from support vector machine (SVM) and k-nearest neighbor (kNN) classification models were shown. Abbreviations: ROC, Receiver operating characteristics.

Of the 3950 breathing sounds, 3159 (80%) were used as training data, and 791 (20%) were used as testing data. First, we evaluated the accuracy, sensitivity, specificity, positive predictive value, and negative predictive value using binary classification: normal and abnormal breathing sounds. MobileNet and Inception_v3 showed the highest values in sensitivity and specificity, with scores of 0.9441 and 0.9414, respectively. ResNet_50 achieved the highest accuracy of 0.9330 and exhibited excellent performance overall across sensitivity, specificity, positive predictive value, negative predictive value, and area under the curve (Table 3). The receiver operating characteristic curves of each classifier are plotted in Fig. 3. When we used three categories, we measured the total accuracy and the “accuracy in abnormal sounds,” which is the accuracy in distinguishing between VS and SS. Among the CNN algorithms, ResNet_50 showed the highest accuracy of 0.9027, and AlexNet showed the highest accuracy of 0.9660 in abnormal sounds (Table 4). Comprehensively, the CNNs showed better classification performances than the machine learning (ML) models (SVM and kNN).

**Table 3:** Evaluation metrics table to classify breathing sounds of 23 patients with a tracheostomy tube: normal and abnormal sounds.

	Input	Name of the classifier	Accuracy	Sensitivity	Specificity	Positive predictive value	Negative predictive value	Area under curve
CNN	Spectrogram	AlexNet	0.9140	0.9401	0.8690	0.9253	0.8936	0.9586
		VGG_16	0.9090	0.9341	0.8655	0.9231	0.8838	0.9527
		ResNet_50	0.9330	0.9381	0.9241	0.9553	0.8963	0.9650
		Inception_v3	0.9317	0.9261	0.9414	0.9647	0.8806	0.9608
		MobileNet	0.9241	0.9441	0.8897	0.9366	0.9021	0.9549
SVM	MFCC(20)	3rd polynomial	0.8306	0.8922	0.7241	0.8482	0.7955	0.9248
		RBF	0.9153	0.9142	0.9172	0.9502	0.8608	0.9681
	MFCC(40)	3rd polynomial	0.8609	0.8703	0.8448	0.9064	0.7903	0.9257
		RBF	0.9102	0.9142	0.9034	0.9424	0.8590	0.9598
kNN	MFCC(20)	k = 3	0.8900	0.9142	0.8483	0.9124	0.8512	0.9098
		k = 5	0.8786	0.8882	0.8621	0.9175	0.8170	0.9159
		k = 7	0.8824	0.8842	0.8793	0.9268	0.8147	0.9369
	MFCC(40)	k = 3	0.9102	0.9341	0.8690	0.9249	0.8842	0.9102
		k = 5	0.8976	0.9102	0.8759	0.9268	0.8495	0.9249
		k = 7	0.8913	0.9042	0.8690	0.9226	0.8400	0.9406

*CNN* convolution neural network; *SVM* support vector machine; *kNN* k-nearest neighbor; *RBF* radial bias function.

**Table 4:** Evaluation metrics table to classify the breathing sounds of 23 patients with a tracheostomy tube into three categories: NS, VS, and SS. We also measured “accuracy in abnormal sounds” to distinguish between VS and SS among the abnormal breathing sounds.

	Input	Name of the classifier	Accuracy	Accuracy in abnormal sounds
CNN	Spectrogram	AlexNet	0.8938	0.9660
		VGG_16	0.8799	0.9509
		ResNet_50	0.9027	0.9489
		Inception_v3	0.9001	0.9461
		MobileNet	0.8951	0.9514
SVM	MFCC(20)	3rd polynomial	0.7295	0.8210
		RBF	0.8217	0.8384
	MFCC(40)	3rd polynomial	0.6738	0.6606
		RBF	0.8306	0.8625
kNN	MFCC(20)	k = 3	0.8458	0.9236
		k = 5	0.8319	0.9169
		k = 7	0.8268	0.9007
	MFCC(40)	k = 3	0.8711	0.9338
		k = 5	0.8496	0.9167
		k = 7	0.8331	0.8985

*NS* normal breathing sounds; *VS* vibrant breathing sounds; *SS* sharp breathing sounds; *CNN* convolutional neural network; *SVM* support vector machine; *kNN* k-nearest neighbor; *RBF*, radial bias function.

## Discussion

There are two major ways to secure an airway. One is intubation, and the other is tracheostomy. After either of those procedures, management, and care of the tube are important. In most cases of intubation, a ventilator, which controls humidity and alarms the status of the tube based on waveforms of respiratory pressure, is supplied^29^. In cases of tracheostomy, on the other hand, many patients maintain spontaneous respiration, and the judgment of obstruction is done by the patient himself or nearby medical staff. However, the judgment of the patient is not always accurate. And medical staff has many other important works to do, so they cannot concentrate only on the respiration of the patient. In addition, this must be done by experienced medical staff. Even in a complex and noisy environment, an objective diagnostic tool on the same level as an experienced medical staff focusing only on respiration is needed, and AI can be considered.

**Figure 4:**
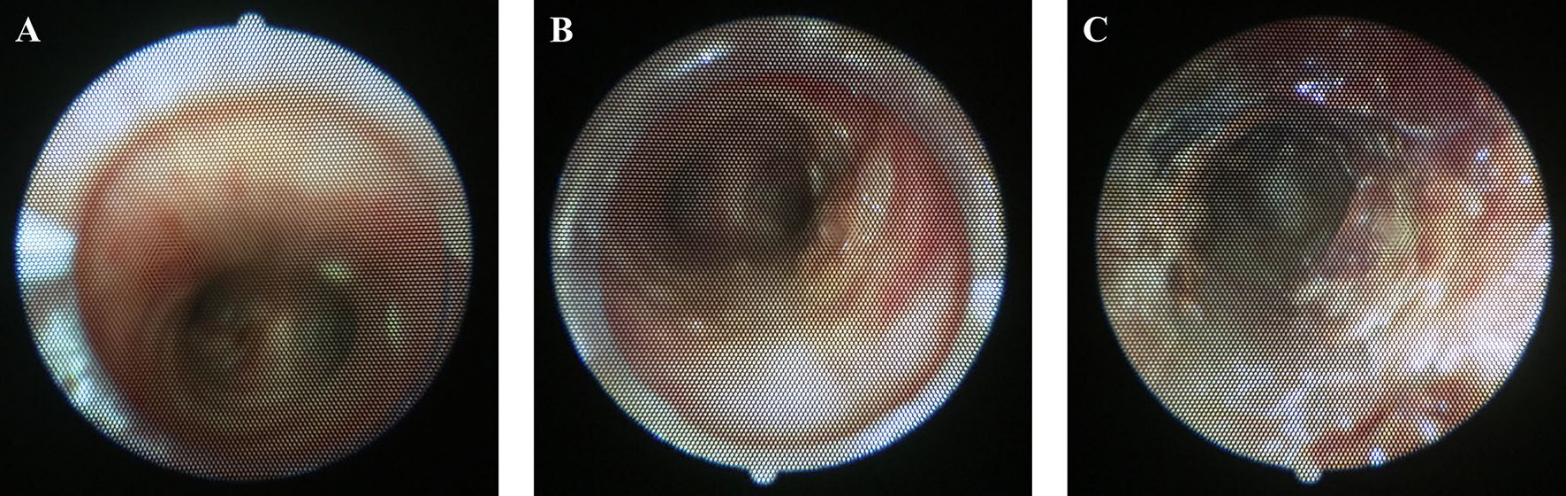
Fiberscope images corresponding to each of the three classifications of breathing sounds. (**A**) Fiberscope image in normal breathing sound. The mucosa of the trachea and carina were well observed. (**B**) Fiberscope image in vibrant breathing sound. Some thick yellow-colored sputum was surrounded by the trachea ring. The surface of the sputum exhibited waves corresponding to breath. After only suction, the participant appealed for improved airway condition. (**C**) Fiberscope image in sharp breathing sound. Blood clots and crust were adhering together, filling the inside of the trachea. They were also filling the inside of the tracheostomy tube. After tube change and suction, the participant appealed comfortable breathing, and the breathing sound was also improved to normal breathing sound.

The fiberscope is considered one of the best methods for assessing the airway. At the start of the study, a fiberscope was used to assess the airway. The standard that used to simply divide breaths into normal and abnormal could be refined further through the fiberscope. The fiberscope revealed that among abnormal breathing sounds, some cases involved movable obstacles that could be resolved with suction, while others involved fixed obstacles that required a tube change. After analyzing the fiberscope images (Fig. 4), this differentiation could be confirmed through the use of a spectrogram, and it served as our starting point. However, it was practically and ethically impossible to keep the fiberscope in the tracheostomy tube during all study periods because the fiberscope itself caused respiratory discomfort for the participants. In some cases, despite the medical staff next to the participants assessing the breathing sounds as VS, little or no sputum was observed through the fiberscope. However, the presence of sputum was confirmed through suction, and the participants themselves also reported improved airway conditions after suction. This limitation arises because the fiberscope typically allows examination only up to the carina, and it is speculated that the discomfort of the patient is not necessarily proportional to the amount of sputum observed. For these reasons, we focused more on analyzing breathing sounds rather than relying solely on the fiberscope.

**Figure 5:**
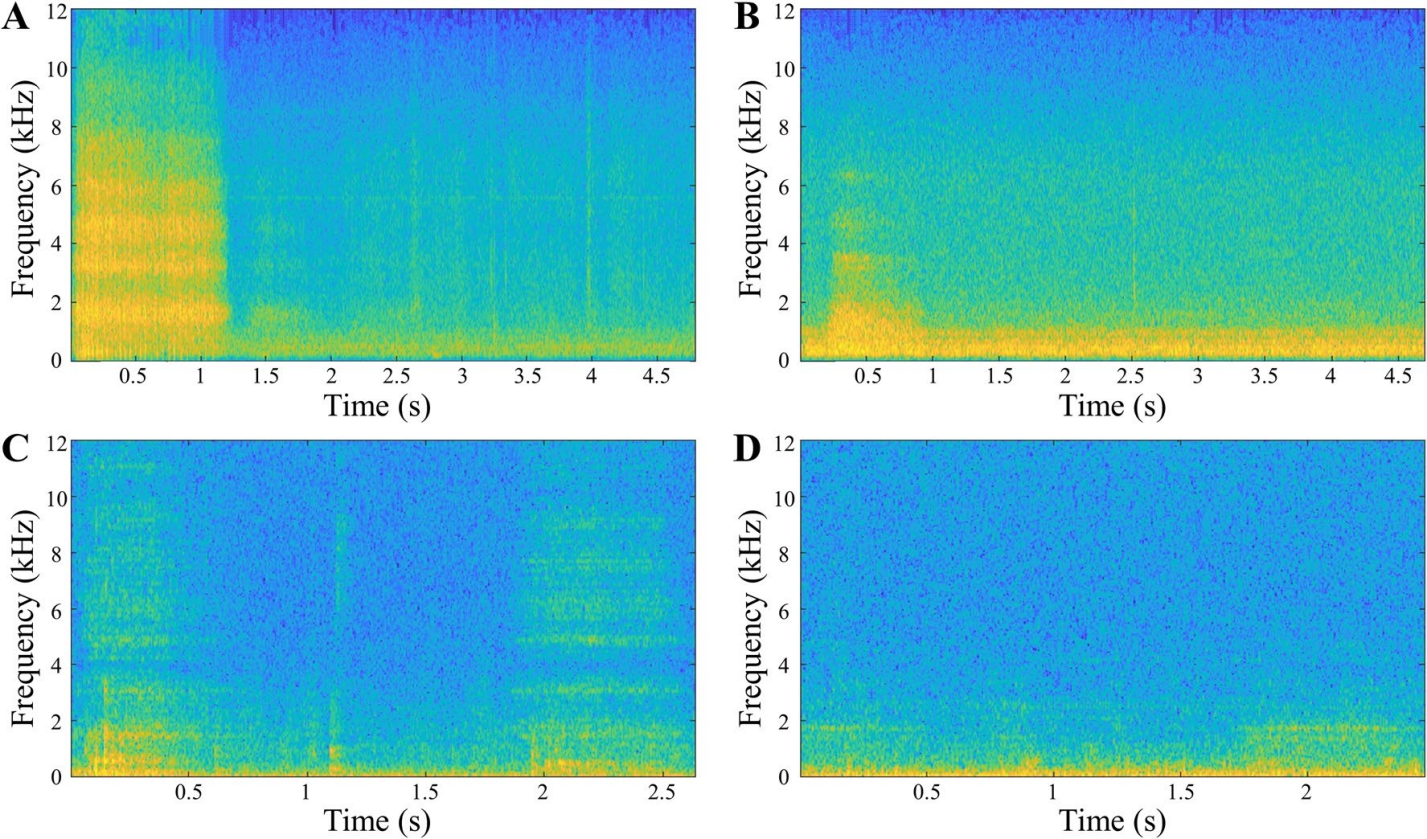
Spectrograms when there are airway problems and when they are resolved. (**A**) Spectrogram when vibrant breathing sounds were heard. Many multiple vertical lines are exhibited for one second. (**B**) Spectrogram 10 min after suction. The length of the vertical line appeared to have decreased. The duration of respiration was also decreased from over one second to under one second. (**C**) Spectrogram when sharp breathing sounds were heard. Multiple horizontal lines exhibited over 2000 Hz. Some acoustic energy was shown above 10,000 Hz. (**D**) Spectrogram after 5 min after tube change and suction. Acoustic energy above 2000 Hz disappeared. Only acoustic energy under 2000 Hz was exhibited during respiration.

Lung sounds are usually classified into two groups: normal and abnormal or adventitious^9^. However, as technology has advanced, there have been recent reports of classifying lung sounds into multiple groups. Chen et al. reported that they achieved up to 98% accuracy in classifying lung sounds into three categories using ResNet: normal, wheezing, and crackle^30^. Borwankar et al. reported that they classified lung sounds into three categories with up to 0.9930 of F1-score using combination of MFCC, Melspectogram and Chroma energy normalized statistics with CNN: normal, chronic, and non-chronic^31^. However, there is no well-known classification for sputum sound. Some reports tried to classify them, but they succeeded only in detecting normal and abnormal breathing sounds^7,32^. We decided to classify the sounds into three categories based on the status of the obstacles, proved by the waveform of the spectrogram: NS, VS, and SS. This difference was expressed not only as the sounds but also as the waveform of the spectrogram. The sound energy of NS was concentrated below 2000 Hz, whereas the sound energy of SS was distributed above 4000 Hz. VS, which involves repeated sound patterns for a very short time, showed the characteristic vertical lines (Fig. 2). These characteristic spectrogram waveforms are the visual basis for the three categories’ classification according to the status of the obstacles. From a clinical point of view, if VS was heard in the tracheostomy tube, most cases could be improved with only suction (Fig. 5A,B). When SS was heard in the tracheostomy tube, some cases might need the change of tube or inner cannula (Fig. 5C,D), while others might only need suction. Even if SS was heard, suction could still be attempted. However, considering the possibility of tube change is necessary because improvement through suction alone is not always guaranteed. Additionally, in some cases, both movable and fixed obstacles can be present in the airway, leading to the occurrence of both VS and SS. We classified those occasions as VS for the reasons mentioned above, additionally because suction is safer and faster than a tube change.

Direct listening was not always accurate for even experts. Although the medical staff initially judged the breathing sounds as normal on-site, many cases were later classified as either VS or SS through the spectrogram waveform of the recording files because both a microphone and the spectrogram allow for more accurate and detailed analysis than the human ear. Even in an environment without soundproofing, such as ICU, numerous recordings were confirmed as VS and SS as a result of using a recording system with a microphone.

When we compared the CNNs with the ML, the CNNs showed much higher accuracy. Since NS and SS have different focused frequency ranges, both CNN and ML could distinguish NS and SS well. However, VS was much better discriminated against by CNN than by ML. Whereas ML judged the type of breathing sounds based on an entire cycle of respiration, CNN could discriminate a temporal element because it analyzed data that had been converted into a spectrogram. Some articles reported that ResNet performed better than any other models in classifying into multiple categories^30^. Our results also showed ResNet achieved the highest accuracy when classifying into three categories. As ResNet usually has advantages to perform with deeper networks, it is thought to achieve better results in classifying multiple categories while effectively minimizing degradation problems^26,30^.

We used polysomnography to assess oxygen saturation, heart rate, blood pressure, respiratory rate, and airflow. We thought that we might find significant correlations between airway obstruction and aerodynamic factors, but we did not, presumably because the aerodynamic factors were controlled more by factors such as pain and sleep than by the degree of airway obstruction. In cases of severe airway obstruction, some aerodynamic factors showed changes, such as respiratory rate and airflow pressure, but those changes were inconsistent.

This study has some limitations. First, these breathing sounds were not obtained in a soundproof facility. Although we excluded sounds that contained severe noise, all our sound data contained some noise. Since the majority of the noise was concentrated below 1500 Hz, we applied filtering based on this criterion. Nevertheless, it was obvious that there were inherent limitations in eliminating all noise, regardless of the extent of noise reduction. However, in reality, it is close to impossible to conduct research in a soundproof facility targeting patients who require ICU care. For this reason, as a workaround, we installed an omnidirectional microphone facing the tube tip and as closely as possible. This allowed us to capture as many breathing sounds as possible in the recordings. If a microphone and tracheostomy tubes are developed as an integrated unit, it is expected that the sensitivity and accuracy of the classification system will further increase. Second, a larger number of the participants is required. The length of the trachea varies significantly among individuals and is influenced by factors such as gender, height, and weight. Therefore, conducting research with a more extensive and diverse sample of individuals is likely to yield more accurate results. In particular, only two of the participants were female. Of course, the breathing sounds from the tracheostomy tube were not much affected by sex differences because the tracheostomy tube was located under the vocal folds and did not process the sound. Nevertheless, the next study should include more female patients.

Despite those limitations, this study is meaningful. First, this is the first study of breathing sounds from a tracheostomy tube. Second, this is the first trial to classify sputum sounds into three categories, confirmed by the spectrogram. Third, CNN classified sputum sounds as the accuracy of our system was very good, better than 90%.

## Conclusion

Classifying breathing sounds into three categories instead of just normal and abnormal can be more useful in identifying several airway problems and determining the appropriate interventions even for caregivers who may not be expert medical staff. This system includes a recording system with a microphone and has an objective basis on the waveform of the spectrogram. If CNN is used, higher accuracy could be acquired.

### Supplementary Information

Supplementary Information.

## Data Availability

The source codes used in this study are available online. The authors will also share sound data from deidentified individual participants in this study with researchers who provide a methodologically viable proposal and have the ability to analyze the data properly. Data-sharing requests can be directed to Younghoon Joo by email immediately following publication. To gain access to the data, requestors will need to sign a data access agreement.
