# Supplementary material for: Breathing sounds analysis system for early detection of airway problems in patients with a tracheostomy tube
Source: Sci Rep. 2023 Nov 29;13:21029. doi: 10.1038/s41598-023-47904-0 (PMC10687247; doi:10.1038/s41598-023-47904-0)
Supplement: Supplementary file 1 — Supplementary Information. [file 41598_2023_47904_MOESM1_ESM.docx]

**Supplementary**

**1.** The conversion process from the audio file to the spectrogram. **2.** The extraction process to Mel frequency cepstral coefficient(MFCC). **3.** The schematic of Mel-filter banks to extract MFCCs. **4.** The examples of utilized MFCCs **5.** Support vector machine (SVM) and k-Nearest neighbor (kNN)

**Supplementary 1. The conversion process from the audio file to the spectrogram**

Each one-cycle breathing sound was segmented into 512 Hamming windows that were overlapped by half the window length. The data in each segmented window were converted into a spectrum through a 512-point discrete Fourier transform (DFT). Because sound in the relatively high-frequency spectrum was unimportant for our purposes, we selected a DFT frequency range of 0–12 kHz. Then, the two-dimensional breathing sound data, i.e., integrated segmented DFT data, were converted into 640 × 360-pixel spectrogram images.

**Supplementary 2. The extraction process to Mel frequency cepstral coefficient**

Mel frequency cepstral coefficients (MFCC) are sound features extracted to facilitate the analysis of sound characteristics referring to human’s auditory system, which is sensitive to difference in lower frequency range. The MFCCs are extracted as following steps.

The sounds for each respiratory cycle were framed with a length of 512-samples, and the frames overlap by half of the frame length, followed by the application of Hann windows. The signals in each frame were converted into spectra using a 512-point discrete Fourier transform (DFT). To extract the MFCC, each DF-transformed spectrum was passed through filter banks to produce the same number of spectra as the number of filters for a frame. We designed filter banks with 20 and 40 filters to extract MFCCs corresponding to the 0–12 kHz frequency range applied to the spectrogram conversion. In the filter bank design, the linear-scale 0–12 kHz frequency range was converted into a mel-scale frequency range using Equation (1), and then the mel-scale range was divided into equal parts based on the band edges (Davis and Mermelstein, 1980; O'shaughnessy, 1987). The designed filter banks are presented in Supplementary 3,

Equation (1) __

The power spectra that passed through each filter underwent discrete cosine transform (DCT) on the log-transformed power spectra to obtain the MFCC, a group of cepstral amplitudes in each frame.

**Reference**

1. Davis, S. & Mermelstein, P. Comparison of parametric representations for monosyllabic word recognition in continuously spoken sentences. *IEEE transactions on acoustics, speech, and signal processing* **28**, 357-366 (1980).

2. O'shaughnessy, D. *Speech communications: Human and machine (IEEE)* (Universities press, 1987).

**Supplementary 3. The schematic of Mel-filter banks to extract MFCCs**

20 filters (A) and 40 filters (B). Because the triangular filter banks were spaced with specific rules but mainly at low frequencies, they reflected more accurately for low-frequency sound analysis. The number of extracted MFCCs corresponded to the number of filters in the Mel-filter banks.


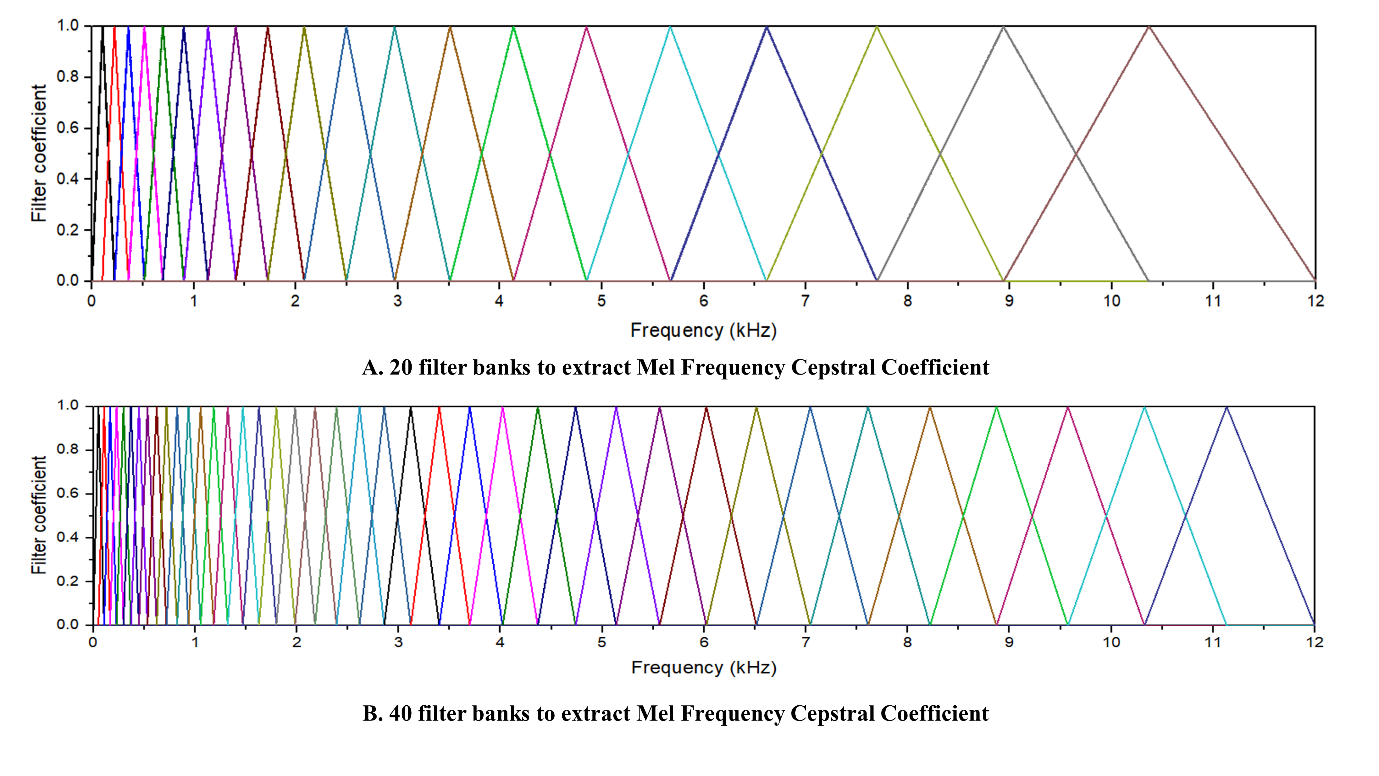


**Supplementary 4. The examples of utilized MFCCs**

Color maps for MFCC(20) and MFCC(40) corresponding to respiratory sounds in Figure 2 are presented: A and B for normal breathing sound (NS); C and D for vibrant breathing sound (VS); E and F for sharp breathing sound (SS), respectively.


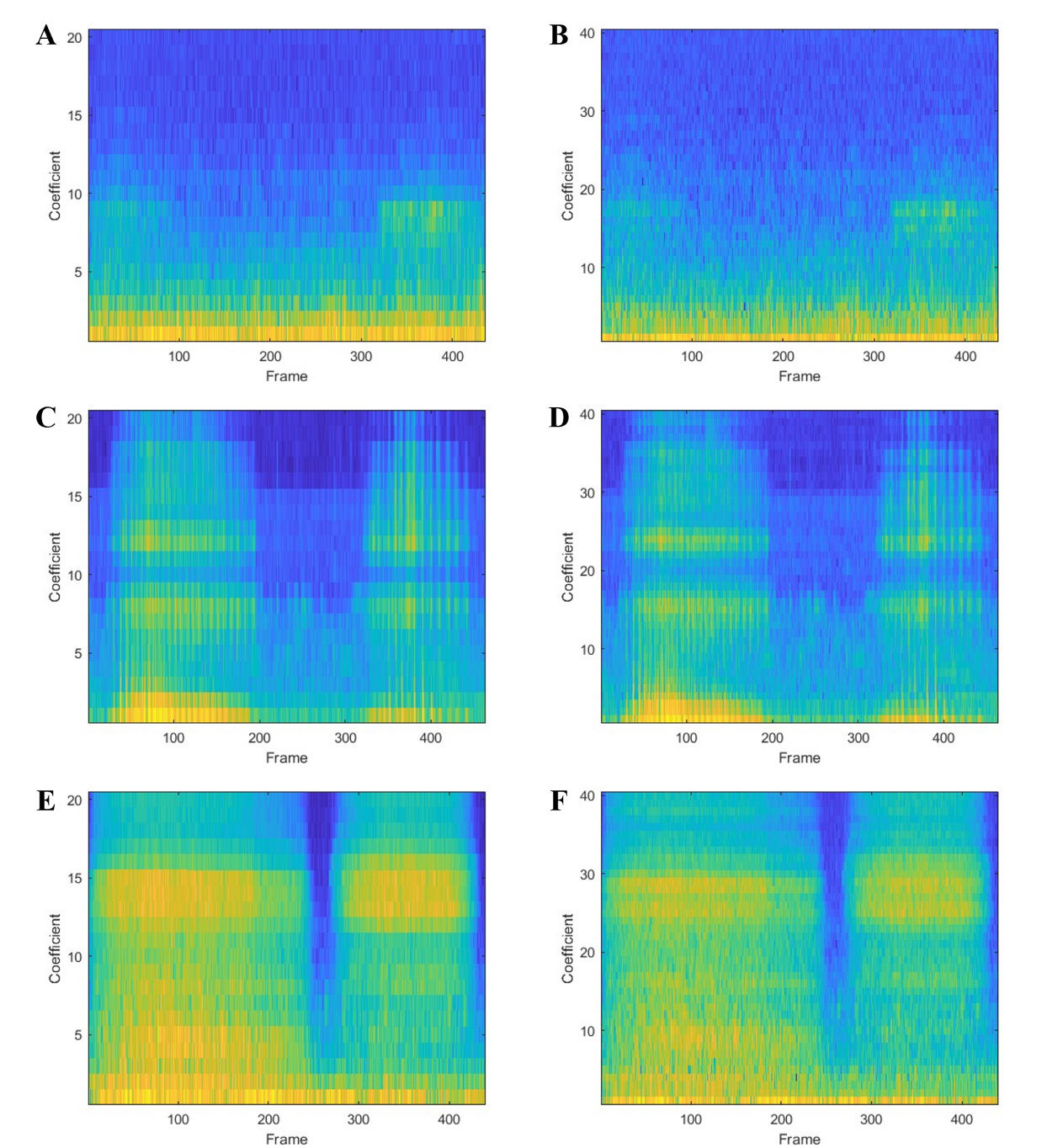


**Supplementary 5. Support vector machine (SVM) and k-Nearest neighbor (kNN)**

4-1 Support vector machine (SVM)

SVM is a classifying algorithm that is trained wih the feature parameters of data in each class and then classifies the test data into hyperplanes (Noble, 2006). Many problems in a variety of data classification fields involve data groups that are not linearly classified, so an SVM applies various kernel functions to help compute the hyperplanes and classify the parameters effectively. SVM-based respiratory classification has been used in fields such as sleep apnea identification (Al-Angari and Sahakian, 2012) and lung disease (Palaniappan et al., 2014). In this study, we used linear, third-order polynomial, and radial-basis (Gaussian) kernel functions for the SVM.

4-2 k-Nearest neighbor (kNN)

kNN is an algorithm that determines the class of test data by finding the ‘k train data space’ nearest to each test data point (Keller et al., 1985). kNN is a widely used algorithm because it is a simple, efficient classifier that does not require separate training. It has frequently been used for breathing sound classification (Haider et al., 2019; Palaniappan et al., 2014). We classified the three breathing sounds by setting the k value to 3, 5, and 7, for NS, VS, and SS, respectively. To find the k nearest data space, the distance between the trained and test data was calculated with a Euclidean algorithm.

**Reference**

1. Noble, W. S. What is a support vector machine? *Nature biotechnology* **24**, 1565-1567 (2006).

2. Al-Angari, H. M. & Sahakian, A. V. Automated recognition of obstructive sleep apnea syndrome using support vector machine classifier. *IEEE Trans Inf Technol Biomed* **16**, 463-468 (2012).

3. Palaniappan, R., Sundaraj, K. & Sundaraj, S. A comparative study of the SVM and K-nn machine learning algorithms for the diagnosis of respiratory pathologies using pulmonary acoustic signals. *BMC Bioinformatics* **15**, 223 (2014).

4. Keller, J. M., Gray, M. R. & Givens, J. A. A fuzzy k-nearest neighbor algorithm. *IEEE transactions on systems, man, and cybernetics*, 580-585 (1985).

5. Haider, N. S., Singh, B. K., Periyasamy, R. & Behera, A. K. Respiratory Sound Based Classification of Chronic Obstructive Pulmonary Disease: a Risk Stratification Approach in Machine Learning Paradigm. *J Med Syst* **43**, 255 (2019).
